# The p66^Shc^ Redox Protein and the Emerging Complications of Diabetes

**DOI:** 10.3390/ijms25010108

**Published:** 2023-12-20

**Authors:** Giuseppina Biondi, Nicola Marrano, Anna Borrelli, Martina Rella, Rossella D’Oria, Valentina Annamaria Genchi, Cristina Caccioppoli, Angelo Cignarelli, Sebastio Perrini, Luigi Laviola, Francesco Giorgino, Annalisa Natalicchio

**Affiliations:** Department of Precision and Regenerative Medicine and Ionian Area, Section of Internal Medicine, Endocrinology, Andrology and Metabolic Diseases, University of Bari Aldo Moro, 70124 Bari, Italymartina.rella@uniba.it (M.R.); rossella.doria@uniba.it (R.D.); valentina.genchi@uniba.it (V.A.G.);

**Keywords:** diabetes, traditional complications of diabetes, emerging complications of diabetes, p66^Shc^, neurodegenerative disease, cognitive disorders, cancer, liver disease, sleep disturbance

## Abstract

Diabetes mellitus is a chronic metabolic disease, the prevalence of which is constantly increasing worldwide. It is often burdened by disabling comorbidities that reduce the quality and expectancy of life of the affected individuals. The traditional complications of diabetes are generally described as macrovascular complications (e.g., coronary heart disease, peripheral arterial disease, and stroke), and microvascular complications (e.g., diabetic kidney disease, retinopathy, and neuropathy). Recently, due to advances in diabetes management and the increased life expectancy of diabetic patients, a strong correlation between diabetes and other pathological conditions (such as liver diseases, cancer, neurodegenerative diseases, cognitive impairments, and sleep disorders) has emerged. Therefore, these comorbidities have been proposed as emerging complications of diabetes. P66^Shc^ is a redox protein that plays a role in oxidative stress, apoptosis, glucose metabolism, and cellular aging. It can be regulated by various stressful stimuli typical of the diabetic milieu and is involved in various types of organ and tissue damage under diabetic conditions. Although its role in the pathogenesis of diabetes remains controversial, there is strong evidence regarding the involvement of p66^Shc^ in the traditional complications of diabetes. In this review, we will summarize the evidence supporting the role of p66^Shc^ in the pathogenesis of diabetes and its complications, focusing for the first time on the emerging complications of diabetes.

## 1. Introduction

Diabetes mellitus is a chronic metabolic disease, the prevalence of which is steadily increasing worldwide. Currently, 537 million adults (20–79 years) are living with diabetes (one in ten), and this number is predicted to rise to 783 million by 2045 [1]. Type 2 diabetes mellitus (T2D), the most common form of diabetes, is characterized by chronic hyperglycemia secondary to the loss of pancreatic beta-cell mass and function associated with insulin resistance. The deficit of functional beta-cell mass is a necessary and early condition for the development of T2D, and it is influenced by both environmental and genetic factors [2]. Indeed, increased levels of glucose and/or fatty acids (FAs) introduced in the diet or due to dysmetabolic conditions (such as obesity and a sedentary lifestyle) raise the metabolic load, causing chronic systemic inflammation, insulin resistance, and increased blood glucose levels. Pancreatic beta-cells can initially compensate for increased glycemia by producing and secreting greater amounts of insulin. Over time, however (depending on the different individual genetic susceptibilities), the high metabolic load promotes beta-cell death and dysfunction [2,3,4,5]. Specifically, chronic hyperglycemia and hyperlipidemia play crucial roles in inducing beta-cell failure, by acting through mechanisms generally defined as gluco-/lipotoxicity. Consequently, insulin secretion is no longer able to compensate for the high blood glucose levels, resulting in an impaired glucose tolerance (IGT), overt hyperglycemia, and T2D. Unlike T2D, type 1 diabetes (T1D, the second most common form of diabetes) is characterized by the autoimmune destruction of pancreatic beta-cells, resulting in an almost absolute insulin deficiency and, therefore, hyperglycemia.

Diabetes is often burdened by disabling comorbidities that reduce the quality of life and life expectancy of the affected individuals. The complications of diabetes are generally described as vascular complications, and classified as macrovascular (e.g., coronary heart disease, peripheral arterial disease, or stroke) or microvascular (e.g., diabetic kidney disease, retinopathy, or neuropathy) [6]. Heart failure is also a common initial manifestation of cardiovascular disease [7], which represents the leading cause of morbidity and mortality in individuals with diabetes. Of note, diabetes and its complications were responsible for 6.7 million deaths in 2021 (one every 5 s) [1]. Although the complications of diabetes occur in both T1D and T2D patients, according to some studies, the juvenile-onset T2D phenotype shows more complications and greater mortality compared to T1D, probably due to its greater association with cardio-metabolic risk factors (such as insulin resistance, obesity, hypertension, and dyslipidemia) [8,9,10,11,12].

Interestingly, in recent years, the advances in diabetes management and the increase in the life expectancy of diabetic patients have made it possible to identify less-recognized and longer-term comorbidities, defined as emerging complications of diabetes [13,14]. Indeed, while deaths from vascular diseases (which once accounted for more than 50% of the deaths in diabetic patients) have declined [15,16,17,18,19], in some countries, cancer and dementia have become the leading causes of mortality in people with diabetes [15,17]. Likewise, diabetes is associated with an increased risk of various cancers, neurodegenerative diseases, and cognitive disorders (especially Alzheimer’s disease and vascular dementia) [20], as well as liver diseases, such as non-alcoholic fatty liver disease (NAFLD), non-alcoholic steatohepatitis (NASH), and liver fibrosis [21,22,23,24,25]. In addition, several studies have recently demonstrated a strong association between diabetes and a broad range of other comorbidities, thus defined as emerging complications of diabetes, which include functional disabilities, affective disorders (especially depression), sleep disorders, and infections (e.g., COVID-19, pneumonia, and foot and kidney infections) [20]. Of note, while traditional complications accounted for over 50% of the hospitalizations of people with diabetes in 2003, they accounted for just 30% of the hospitalizations in 2018 in England [26], probably giving way to emerging complications. These data confirm the changing nature of diabetes complications in recent years and highlight the need to review diabetes management to improve diabetic patients’ quality and expectancy of life.

The p66^Shc^ protein is the largest of three protein isoforms (p66^Shc^, p52^Shc^, and p46^Shc^) encoded by the proto-oncogene *ShcA* (*Src collagen homologue A*) [27]. *ShcA*-family isoforms are adaptor proteins capable of recruiting different signaling molecules and are involved in several cellular pathways (e.g., proliferation, growth, and survival). Unlike p52^Shc^ and p46^Shc^, which are consistently expressed in nearly all cell types, p66^Shc^ expression levels vary depending on tissues, aging, and pathological conditions. Furthermore, it has peculiar functions, mainly due to its specific collagen homologue 2 (CH2) domain at its N-terminal, containing serine phosphorylation sites. In particular, through the phosphorylation of the serine at position 36 of the amino acid sequence (Ser36), p66^Shc^ negatively interferes with glucose metabolism and modulates oxidative stress (acting as both a redox sensor and an enzyme), inducing apoptosis in several cell types and playing a role in cellular aging [28,29,30,31,32]. It has been demonstrated that various stressor stimuli typical of the diabetic milieu (such as oxidative stress, cytokines, hyperglycemia, and FAs) can induce p66^Shc^ protein expression and/or activation in several cell types. In addition, p66^Shc^ is already known to be involved in various types of organ and tissue damage under diabetic conditions [33,34,35,36,37]. Although its role in the pathogenesis of diabetes remains controversial, there is strong evidence for the involvement of p66^Shc^ in the traditional complications of diabetes.

In this review, we will summarize the evidence on the role of p66^Shc^ in the onset and progression of diabetes and its complications, focusing for the first time on the emerging complications of diabetes.

## 2. The Role of p66^Shc^ in Diabetes and Its Traditional Complications

### 2.1. The Role of p66^Shc^ in Pancreatic Beta-Cell Failure and Insulin Resistance

The role of p66^Shc^ in diabetes has been widely studied in recent years. Possible involvement of p66^Shc^ in diabetes was suggested by a study that identified ShcA proteins to be among the most discriminating urinary biomarkers of the transition from prediabetes to T2D [38]. Specifically, the ShcA protein levels significantly decreased from controls to prediabetic patients, while they increased from prediabetes to T2D [38,39]. In these patients, the combination of ShcA protein levels and BMI values showed a sensitivity of 100% as predictive biomarkers of prediabetes, while the predictive power of HbA1c did not exceed 50%. Since ShcA proteins may physiologically act as scaffold proteins for insulin signaling in many cells, and chronic hyperinsulinemia can negatively affect this pathway, the reduction in urinary ShcA protein levels observed in prediabetic patients could be due to the hyperinsulinemia typical of prediabetes [39]. Nevertheless, the specific contribution of p66^Shc^ to this reduction has not been investigated.

Unlike what has been observed in prediabetes, the p66^Shc^ levels have been found to be increased in the peripheral blood mononuclear cells (PBMCs) of patients with diabetes, as well as in the serum and placenta of patients with gestational diabetes mellitus [40,41], supporting the hypothesis that p66^Shc^ levels are increased in diabetes. Of note, both excess of FAs and obesity, which are leading risk factors for diabetes, can specifically increase the levels of p66^Shc^ protein, as well as its activation through phosphorylation in Ser36, in cells and tissues involved in glucose homeostasis, such as insulin-secreting pancreatic beta-cells [29,30,42], adipocytes [43], and hepatocytes [44], thus causing cellular stress, apoptosis, cellular dysfunction, and insulin resistance, therefore favoring the pathogenesis of diabetes.

In particular, the observation that p66^Shc^ can mediate pancreatic beta-cell failure under gluco-/lipotoxic conditions strongly supports the hypothesis that p66^Shc^ contributes to the onset and progression of diabetes. In fact, increased p66^Shc^ protein expression and phosphorylation at Ser36, together with increased apoptosis levels, were found in human and mouse islets and in rat insulin-secreting INS-1E cells chronically exposed to high levels of glucose or saturated fatty acids (SFAs). P66^Shc^ protein expression and function were also found to be elevated in islets from high-fat-diet (HFD)-fed mice compared to mice fed a standard diet, and in pancreatic islets of obese patients compared to lean subjects [30]. In addition, high glucose or SFA-induced apoptosis was abrogated in islets from *p66^Sh^*^c^-knockout mice and following a *p66^Shc^* knockdown in INS-1E cells. Conversely, beta-cell apoptosis was increased by the overexpression of *p66^Shc^*, but not in a phosphorylation-defective *p66^Shc^* mutant (Ser36 to Ala), thus suggesting a specific role of Ser36 phosphorylation in these events [30]. Interestingly, p66^Shc^ appeared to mediate SFA-induced apoptosis more than high glucose-induced apoptosis in pancreatic beta-cells. In fact, while *p66^Shc^* knockdown completely abrogated SFA-induced apoptosis, glucose-induced beta-cell death was inhibited by only 50%. SFA-mediated p66^Shc^ activation is also involved in the beta-cell insulin resistance induced by chronically elevated SFA levels. In particular, SFA-mediated p66^Shc^ activation led to the activation of S6 kinase (S6K) and the inhibition of insulin receptor substrate-1 (IRS-1), resulting in insulin signaling inhibition and a reduction in insulin’s stimulatory effects on its own biosynthesis and secretion [29].

Studies on *p66^Shc^* deletion in vitro in cell types other than pancreatic beta-cells have provided further data supporting the possible role of p66^Shc^ in the pathogenesis of diabetes, mainly due to its insulin-signaling repressor activity [45]. Indeed, it has been demonstrated that p66^Shc^ can mediate cellular insulin resistance by interacting with S6K and IRS-1 to form a complex that is able to inhibit insulin signaling under lipotoxic conditions in endothelial cells and adipocytes [43,46]. According to these results, the knockdown of *p66^Shc^* increased the insulin sensitivity in several cell types in vitro (including liver, kidney, and muscle cells) [47] and enhanced the glucose uptake in HeLa cells and murine embryonic fibroblasts [48]. Additionally, *p66^Shc^* deletion resulted in the upregulation of glucose transporters (GLUT 1 and 3) and increased basal glucose transport in myoblasts, whereas its overexpression produced the opposite effects [33].

Despite the in vitro results, the data on the ability of p66^Shc^ to affect glucose homeostasis in lean or obese whole-genome-knockout mice for the *p66^Shc^* gene (*p66^Shc-/-^*) are controversial (reviewed in [45]). Indeed, while Ranieri SC et al. identified p66^Shc^ as a major mediator of insulin resistance and T2D in leptin-deficient (*ob/ob*) mice [43], Ciciliot S et al. demonstrated that reduced p66^Shc^ levels were protective against obesity, without preventing insulin resistance, ectopic fat accumulation, or glucose intolerance, in the same mouse model and in humans [49]. The modified gut microbiome found in *p66^Shc-/-^* mice may, in part, explain the lack of improvement in HFD-induced metabolic dysfunction, despite a consistently leaner phenotype, as dysbiosis could be responsible for insulin resistance independently of weight gain [50]. Conversely, a study performed on a different *p66^Shc-/-^* mouse model, named ShcL, demonstrated that reduced p66^Shc^ levels improved insulin sensitivity in adipocytes, muscles, and the liver without preventing diet-induced obesity [47]. These controversial results suggest the need to explore the p66^Shc^ action in different mice models and to develop tissue-specific and inducible *p66^Shc-/-^* mice to identify the specific role of p66^Shc^ in various tissues, as well as for different developmental stages or timings of exposure to environmental stresses (e.g., HFD). In fact, carrying out experiments in mice at different ages and with different durations of the HFD regimens can lead to conflicting results [45], which are sometimes difficult to reconcile.

With regards to T1D, studies have reported that *p66^Shc-/-^* mice were not resistant to streptozotocin-induced diabetes. In fact, after streptozotocin injection, both *p66^Shc-/-^* and wild-type mice showed the same increase in blood glucose levels [51,52].

In conclusion, although robust evidence endorses the involvement of p66^Shc^ in pancreatic beta-cell failure and loss, the findings on insulin sensitivity, glucose tolerance, glucose uptake, and metabolism have not been conclusive, questioning the role of p66^Shc^ in diabetes induction and progression.

### 2.2. The Role of p66^Shc^ in Traditional Complications of Diabetes

**Figure 1:**
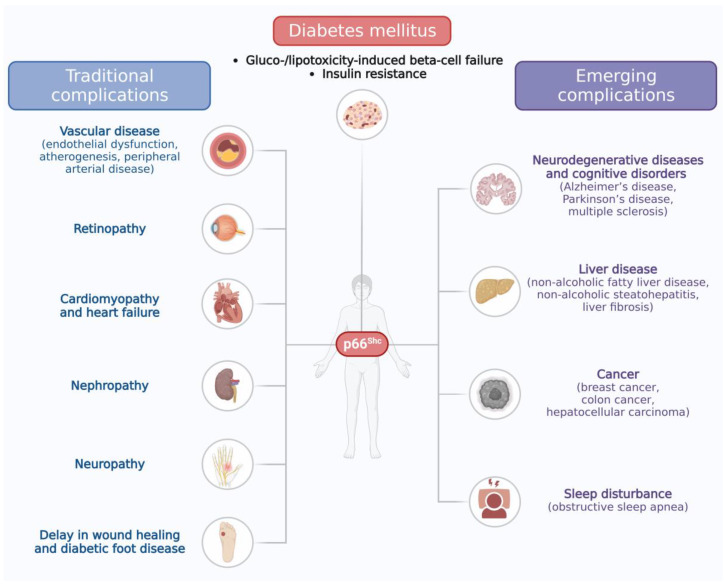
The p66^Shc^ protein has been recognized to be involved in gluco/lipotoxicity-induced beta-cell failure and insulin resistance, which represent the two hallmarks of type 2 diabetes pathogenesis. On the other hand, the involvement of p66^Shc^ in the onset of type 1 diabetes has not been established. In addition, p66^Shc^ has been demonstrated to play an important role in many traditional (on the left) and emerging (on the right) complications of diabetes.

Several pieces of evidence have confirmed the role of p66^Shc^ in promoting the traditional complications of diabetes (Figure 1). Numerous studies have shown that diabetic *p66^Shc-/-^* mice are protected from the onset of micro- and macro-vascular complications compared to their wild-type counterparts [51,52,53,54,55,56,57]. It has been shown that *p66^Shc^* gene deletion increases the endothelial expression of antioxidant enzymes, alleviates hyperglycemia-induced oxidative stress, and prevents diabetes-induced endothelial dysfunction in streptozotocin-treated mice [52]. Concordantly, p66^Shc^ was found to be involved in major diabetes-related microangiopathies, namely nephropathy, retinopathy, and neuropathy. In particular, it has been demonstrated that high glucose-mediated *p66^Shc^* overexpression induces podocyte apoptosis through the Notch-PTEN-PI3K/Akt/mTOR pathway, thus suggesting its possible involvement in diabetic kidney disease pathogenesis [58]. Accordingly, the renal expression of *p66^Shc^* was increased in diabetic rats, while the knockout of *p66^Shc^* reduced glomerular injury and restored renal microvascular responses. Moreover, the epigenetic inhibition of *p66^Shc^* ameliorated diabetic nephropathy in mice [59,60]. A hyperglycemia-induced increase in *p66^Shc^* expression was also found in human retinal endothelial cells and retinal pigment epithelial cells. Increased *p66^Shc^* expression resulted in functional damage and/or apoptosis in these cells, thus contributing to the development of diabetic retinopathy [61,62]. Diabetic autonomic neuropathy (DAN) is a further diabetes complication suggested to exert deleterious effects on bone marrow innervation and the mobilization of hematopoietic stem/progenitor cells (HSPCs), also known as HSPC mobilopathy. The latter effect can impair the recovery after vascular ischemia, thus increasing the risk of cardiovascular diseases related to DAN. Interestingly, DAN increased the *p66^Shc^* expression in the bone marrow cells of T1D and T2D diabetic mice models and in the PBMCs of diabetic patients, while a *p66^Shc^* deletion in diabetic mice prevented these DAN-mediated deleterious effects [63]. In particular, it has been shown that overactive p66^Shc^ coupled impaired myelopoiesis with the HSPC mobilopathy that occurs under diabetes. Concordantly, in diabetic patients, *p66^Shc^* gene expression in PBMCs was correlated with myelopoiesis and increased in the presence of peripheral arterial disease (PAD), a condition characterized by the defective traffic of HSPCs [64], while the deletion of *p66^Shc^* in hematopoietic cells reduced aberrant myelopoiesis and rescued postischemic HSPC mobilization and homing under diabetic conditions [65]. Interestingly, p66^Shc^ has been found to be responsible for vascular hyperglycemic memory, that is, the persistence of hyperglycemia-induced vascular functional damage, despite the restoration of euglycemia in diabetic patients. This occurs because the normalization of glucose levels cannot reverse the epigenetically regulated activation of *p66^Shc^* and the concomitant increase in reactive oxygen species (ROS) in endothelial cells. In contrast, the silencing of *p66^Shc^* reduces ROS production, restores endothelium-dependent vasorelaxation, and attenuates endothelial cell apoptosis [66]. Likewise, in diabetes-associated atherosclerosis, the epigenetically sustained *p66^Shc^* expression in plaque macrophages drives the hyperglycemic memory typical of this pathological condition, while the inhibition of *p66^Shc^* abolishes the sustained pro-atherogenic phenotype [67]. P66^Shc^ has been found to also play a direct role in cardiomyopathy. Indeed, as already described for endothelial dysfunction and atherogenesis under diabetic conditions, epigenetic changes in the *p66^Shc^* promoter are responsible for the persistence of cardiomyopathy, despite intense glycemic control. Concordantly, *p66^Shc^* expression has been found to be persistently increased through epigenetic regulation in the heart of diabetic mice and to be associated with oxidative stress, myocardial inflammation, and left ventricular dysfunction. Also in this case, *p66^Shc^* silencing blunted oxidative stress and restored cardiac function in diabetic mice [68]. A *p66^Shc^* gene deletion was also found to prevent diabetes-induced cardiac stem cell aging and heart failure [56]. Finally, it has been demonstrated that a *p66^Shc^* deletion in mouse models accelerated diabetic wound healing by reducing advanced glycation, oxidative stress, and tissue damage, while promoting granulation tissue development and vascularization, inhibiting the anti-healing epithelial pathway, and modulating the vascular response to ischemia. The deletion of *p66^Shc^* also significantly protected against diabetes-related ischemic damage in limb muscles, a factor contributing to diabetic foot disease [55].

Of note, in a study on diabetic patients (5–6 years of follow-up), the p66^Shc^ expression levels in PBMCs were not associated with prevalent diabetic complications at baseline. However, they predicted the onset of new complications at follow-up, especially macroangiopathy [69].

Confirming the involvement of p66^Shc^ in diabetes complications, several drugs, and substances that target p66^Shc^ or its upstream and downstream molecules have beneficial effects against the complications associated with diabetes (as reviewed in [70]).

On the other hand, since the molecular pathways involved in p66^Shc^ action appear to be different based on the cell type and not yet fully understood, it is necessary to further study and elucidate the intracellular role and the pathway of p66^Shc^ before using it as a therapeutic target [70].

Although it is known that stressor stimuli typical of the prediabetic or diabetic milieu (especially FAs and hyperglycemia) can induce p66^Shc^ activation in many cell types and tissues, thus contributing to the onset of diabetes and its complications, based on the available results, it is not yet clear whether the main trigger of p66^Shc^ is excess glucose or FAs. As obesity represents the main risk factor of diabetes, lipotoxicity may act as a triggering factor of p66^Shc^ in the pathogenesis of diabetes [29,30,42,43,44]. On the other hand, elevated blood glucose has been more widely regarded as a trigger of p66^Shc^ in diabetes complications that arise in a more advanced stage of the disease, when hyperglycemia is overt [60,61,62,63,64,65,66,67,68,69]. Despite this, for example, FAs have been shown to activate p66^Shc^ in endothelial or renal cells, thus contributing to the onset of diabetic complications [46,71,72,73,74]. Therefore, it is possible that FAs and excess glucose may contribute in different ways to activating p66^shc^ depending on several variables (e.g., cell type, biological event assessed, stage of diabetes).

## 3. The Role of the p66^Shc^ Protein in Emerging Complications of Diabetes

### 3.1. Liver Disease

Non-alcoholic or metabolic dysfunction-associated fatty liver disease (NAFLD or MAFLD, respectively) is a condition characterized by excessive lipid accumulation in the liver. When severe, it can progress to a kind of non-viral and non-alcoholic hepatitis called non-alcoholic or metabolic dysfunction-associated steatohepatitis (NASH or MASH, respectively), characterized by hepatic inflammation and necrosis, which can result in advanced fibrosis, hepatic cirrhosis, and/or hepatocellular carcinoma [75,76].

T2D is an important risk factor for NAFLD and can accelerate its progression in NASH and advanced fibrosis [77]. Several factors are implicated in the etiology and progression of NAFLD in people with T2D, including hyperglycemia, dyslipidemia, obesity, and insulin resistance [78]. They can impair intrahepatocellular lipid metabolism by increasing the uptake of exogenous FAs in the liver, hepatic lipogenesis, and de novo lipogenesis, while reducing lipolysis, oxidation, and the secretion of FAs [24,79,80,81]. The combination of other factors, such as inflammation, endotoxins, adipokines, hepatokines, mitochondrial dysfunction, oxidative stress, and hepatocyte apoptosis, contributes to the progression of NAFLD into NASH, and finally, into cirrhosis [82].

The total and phosphorylated p66^Shc^ (Ser36) protein and its mitochondrial translocation were found to be increased in the liver of HFD-fed mice. Interestingly, the inactivation of hepatic *p66^Shc^* attenuated the mitochondrial fragmentation and the consequent oxidative stress, preventing HFD-induced hepatic insulin resistance and hyperglycemia [83]. Despite these results, a recent study demonstrated that whole-genome *p66^Shc-/-^* mice are not protected from hepatic insulin resistance and lipid accumulation induced by overfeeding [49]. On the other hand, p66^Shc^ mRNA and protein expression levels were found to be significantly elevated in human NAFLD liver samples compared to normal liver specimens. In addition, among patients with NALFD, those affected by NASH showed significantly higher hepatic levels of both total and phosphorylated (Ser36) p66^Shc^ [84]. Interestingly, the p53/p66^Shc^ pathway could play a role in regulating the progression of NASH, since the *p53* knockout suppressed p66^Shc^ signaling and, at the same time, it decreased hepatic lipid peroxidation and the number of apoptotic hepatocytes and ameliorated the progression of nutritional steatohepatitis [84]. The role of p66^Shc^ in the pathogenesis of NALFD is further supported by a study demonstrating that carnosic acid, a plant-derived compound with antioxidant and anti-apoptotic effects, can prevent the pro-apoptotic effects of the SFA palmitate in human liver cells L02 and reduce the NAFLD incidence in rats fed with HFD by inhibiting the miR-34a/SIRT1/p66^Shc^ pathway [85]. Similarly, isosteviol, a derivative of stevioside, prevents FA-/HFD-induced hepatic injury by modulating PKC-β/p66^Shc^/ROS and endoplasmic reticulum stress pathways in primary rat hepatocytes in vitro and in a rat NAFLD model in vivo [44]. Recent findings have indicated that hepatocyte senescence can contribute to hepatic steatosis in NAFLD, and it may be caused by the upregulation of *p66^Shc^* expression. Concordantly, increased hepatic p66^Shc^ protein levels were correlated with an enhanced expression of the senescence marker p21 and mirrored the degree of disease severity in NAFLD patients [84,86].

Finally, since several studies have demonstrated the involvement of p66^Shc^ signaling in liver fibrosis, its role in the progression of liver fibrosis under diabetic conditions should not be excluded [87,88,89].

### 3.2. Cancer

Increasing evidence suggests that patients with diabetes, particularly T2D, are characterized by an increased risk of developing different types of cancer (especially hepatocellular, pancreatic, gallbladder, colorectal, breast, and endometrial cancers) [90,91]. Hyperglycemia, hyperinsulinemia, excess of FAs, and chronic low-grade inflammation typical of T2D, could be possible mechanisms responsible for the rise in the cancer risk in diabetic patients [92,93,94]. Each of them can lead to greater ROS production and the worsening of oxidative stress [95], which is known to be a biological event that is able to trigger or enhance the tumorigenic process [93], especially when it occurs in tumor suppressor genes [96].

In this scenario, the abnormal activation of p66^Shc^ that occurs in diabetes (sustained by hyperglycemia, hyperinsulinemia, an excess of FAs, and chronic low-grade inflammation) enhances oxidative stress, representing a mechanism in the pathogenesis of cancer and possibly explains the strong correlation between cancer and diabetes. This effect might appear somewhat controversial since p66^Shc^-generated oxidative stress plays an important role in the removal of damaged cells via ROS-induced apoptosis [97,98]. Moreover, p66^Shc^ activation halts the Ras-MAPK pathway, reducing the cell proliferation and migration rate [97,98]. Thus, p66^Shc^ activation can even protect tissues from uncontrolled cancer cell expansion by reducing proliferation and facilitating the elimination of damaged cells. Nevertheless, when unrestrained, p66^Shc^-induced oxidative stress can promote the accumulation of mutations in oncogenes or tumor suppressor genes, nullifying the anti-proliferative and pro-apoptotic role of p66^Shc^ and, thus, triggering tumorigenesis [97]. In addition, ROS produced by p66^Shc^ may act as second messengers of cell proliferation [97]. Accordingly, it has been demonstrated that *p66^Shc^/p53* double-knockout animals are protected from the oncogenic effect observed in *p53* single knockouts [99]. In line with these observations, several studies have demonstrated that *p66^Shc^* is overexpressed in various cancers, including those most linked to diabetes (i.e., breast cancer, hepatocellular carcinoma, and colon cancer) [100], and it is involved in different key elements of tumorigenesis such as cell proliferation, cell migration, and cell adhesion [97].

In particular, the overexpression of p66^Shc^ is associated with cancer development and metastasis [101,102,103], in part due to the activation of the AKT pro-survival pathway, in breast cancer cell lines [104]. Accordingly, it has been demonstrated that the functional inactivation of p66^Shc^ inhibits the epithelial–mesenchymal transition in breast cancer cells, limiting cancer metastasis [105]. In addition, in human breast cancer cells, *p66^Shc^* overexpression increased the activation of Rac1, which has been implicated in cancer initiation, progression, invasion, and metastasis [106]. Similarly, in colon cancer, a high expression of *p66^Shc^* enhanced cancer cell survival rates by activating the PI3K/AKT pathway [107], and it was correlated with a poor cancer prognosis [108]. In hepatocellular carcinoma, *p66^Shc^* expression was found to be increased [109], and this overexpression was coupled with STAT3 activation, which is a positive regulator of cell growth and tumor progression [110]. Accordingly, in this kind of cancer, low p66^Shc^ levels have been associated with better survival [111].

These data suggest that p66^Shc^ activation could represent a shared pathogenetic mechanism in the development of T2D and cancer, and this could explain the increased risk of developing different types of cancer in diabetic patients. Nevertheless, in order to endorse this hypothesis, further studies are needed to confirm p66^Shc^’s involvement in the pathogenesis of both T2D and cancer.

### 3.3. Neurodegenerative Diseases and Cognitive Disorders

Accumulating evidence has demonstrated a ~1.4–2-fold increase in the risk of developing progressive cognitive impairments and neurodegenerative diseases in T2D patients [112,113]. ROS accumulation, oxidative stress, and mitochondrial dysfunction are considered the main drivers of diabetes-related brain damage, including neurodegenerative complications and cognitive disorders [114,115]. In particular, glucose autoxidation, lipid peroxidation, and the decreased activity of antioxidant enzymes increase ROS production in the diabetic brain [114,115] leading to mitochondrial structural and functional alterations. These mechanisms activate the apoptotic cascade by triggering the release of cytochrome c, promoting neuronal apoptosis and, therefore, the development of cognitive or neurodegenerative disorders [116,117].

As previously mentioned, p66^Shc^ is a key regulator of ROS production and mitochondrial function, and so may also play a role in the pathogenesis of diabetic neurodegenerative or cognitive complications. Accordingly, it has been demonstrated that a *p66^Shc^* knockout results in a reduction in inflammation and oxidative stress markers, with an improvement in the cognitive decline in diabetic mice, thus endorsing p66^Shc^’s involvement in oxidative stress-mediated brain damage in mouse models of diabetes [115].

In Alzheimer’s disease (AD), diabetes-related oxidative stress in the brain promotes the accumulation of neurotoxic Aβ in synaptic mitochondria, inhibiting mitochondrial respiration and biogenesis, impairing mitochondrial electron transport chain function, and resulting in the overproduction of ROS, which, in turn, increases the processing of the amyloid-beta precursor protein (APP) for Aβ deposition [116]. Several studies have demonstrated that p66^Shc^ mediates the toxicity induced by neurotoxic Aβ deposition. Indeed, the treatment of human SH-SY5Y neuroblastoma and neuronal PC12 cells with exogenous Aβ results in the increased phosphorylation of p66^Shc^ at Ser36, ROS accumulation, and cell death, partially in a JNK-dependent manner. Accordingly, the overexpression of a phosphorylation-defective *p66^Shc^* mutant (Ser36 to Ala) reduces the Aβ-induced intracellular ROS levels and protects cells against Aβ-induced death [118]. On the other hand, the increased expression and activation of p66^Shc^ in central nervous system cells promotes a metabolic shift from mitochondrial-dependent oxidative phosphorylation to aerobic glycolysis, thus increasing the sensitivity to Aβ toxicity [119]. In contrast, the genetic ablation of *p66^Shc^* in PSAPP transgenic mice, an established AD mouse model of Aβ-amyloidosis, reverses cognitive deficits by improving mitochondrial dysfunction and oxidative damage. Interestingly, these beneficial effects are not associated with alteration of Aβ levels, confirming a role for p66^Shc^ in mediating Aβ-induced toxicity, rather than Aβ expression and accumulation [120].

P66^Shc^-mediated oxidative stress and mitochondrial dysfunction are also involved in the pathogenesis of Parkinson’s disease (PD), another neurodegenerative disorder generally associated with diabetes [121]. It has recently emerged that PD is associated with mutations and, therefore, alterations in the expression of genes coding for PTEN-induced putative kinase 1 (Pink1), Parkin, and DJ-1, which are proteins leading to impaired mitophagy and the accumulation of dysfunctional mitochondria [121]. It was demonstrated that *Pink-1*-deficient cells from PD patients showed hyperphosphorylation of p66^Shc^ as well as a reduction in mitochondrial antioxidant enzymes under oxidative stress conditions [122]. It is interesting to observe that the dysregulation of these genes has also been found in *db/db* mice and HFD-fed mice, thus suggesting an involvement of p66^Shc^-mediated oxidative stress and mitochondrial dysfunction in diabetic brains developing PD [122].

Even in multiple sclerosis (MS), a neurodegenerative autoimmune disease with a higher incidence in patients with T2D, p66^Shc^-mediated mitochondrial oxidative stress may contribute to neuronal damage [123,124]. Indeed, in mouse models of experimental autoimmune encephalomyelitis, a demyelinating autoimmune disease that closely resembles human MS, the deletion of *p66^Shc^* determined a delayed onset and a lower severity of the disease. In particular, it has been suggested that p66^Shc^ activation under oxidative stress conditions generates, in turn, hydrogen peroxide by reacting with cytochrome c, which induces the oxidation of the mitochondrial permeability transition pore and mitochondrial swelling, thus triggering neuronal apoptosis [124]. Therefore, it is possible that diabetes-induced oxidative stress may contribute to the activation of p66^Shc^ and, thus, to the pathogenesis of this neurodegenerative complication.

In conclusion, several studies have demonstrated a direct involvement of p66^Shc^ in the development of dementia in diabetes patients and have suggested a crucial role of p66^Shc^ in oxidative stress and mitochondrial dysfunction in neurodegenerative diseases such as AD, PD, and MS. Since most of these mechanisms are common to diabetes pathogenesis, it is likely that p66^Shc^ plays a causal role in the development of these neurodegenerative disorders in diabetic patients.

### 3.4. Sleep Disturbance

There is a strong association between sleep disorders and the development of T2D. Sleep disorders typically affect the quality, amount, and timing of sleep, leading to impaired daytime functioning and distress. There is probably a bidirectional implication between sleep disorders and T2D, as poor sleep quality could increase the risk of T2D incidence and T2D patients with sleep disturbances show a worse prognosis. Indeed, sleep alterations are associated with decreased insulin sensitivity, the overactivation of inflammatory pathways, impaired brain glucose metabolism, hyperactivation of the hypothalamus–pituitary–adrenal axis with an overproduction of cortisol, suboptimal self-care (i.e., lower medication adherence), and impaired decision making (i.e., unhealthy diet and sedentary behavior) [125,126].

In particular, obstructive sleep apnea (OSA) is characterized by nocturnal intermittent hypoxemia and is linked to oxidative stress and glucose metabolism dysfunction. Since p66^Shc^ plays a key role in regulating oxidative stress and glucose metabolism, Lyu X et al., investigated the expression of *p66^Shc^* in the PBMCs of patients with OSA and its association with polysomnographic parameters. They found that the p66^Shc^ mRNA and protein levels in PBMCs were significantly higher in OSA patients compared to controls and that the *p66^Shc^* mRNA levels were positively correlated with the hypopnea index, oxygen desaturation index, percentage of total sleep time with an oxygen saturation below 90%, and Epworth sleepiness scale in OSA patients [127]. Despite this, whether p66^Shc^ is involved in the pathogenic process of OSA and whether it is related to the severity of intermittent hypoxia remain unknown [127].

Although p66^Shc^’s involvement in sleep disorders needs to be investigated, p66^Shc^’s correlation with hyperglycemia, inflammatory pathways, impaired glucose metabolism, and oxidative stress is known. Since these mechanisms are shared by T2D and sleep disorders, an involvement of p66^Shc^ in the pathogenesis of the latter under diabetic conditions is conceivable [70].

## 4. Conclusions

The advances in diabetes management and the increase in the life expectancy of diabetic patients have made it possible to identify less-recognized and longer-term diabetes comorbidities, defined as emerging complications of diabetes [13,14,20]. Likewise, diabetes is associated with an increased risk of various neurodegenerative diseases, cognitive disorders, liver diseases, and cancers, as well as functional disabilities, affective disorders, sleep disorders, and infections [13,14,20,21,22,23,24,25,91]. Apparently, the available and upcoming anti-diabetes drugs are not able to challenge these emerging complications.

P66^Shc^ is a redox protein that plays a role in oxidative stress, apoptosis, glucose metabolism, and cellular aging [28,29,30,32,34]. It is involved in various types of organ and tissue damage under diabetic conditions, and, there is strong evidence regarding its involvement in the traditional complications of diabetes (Figure 1). Concordantly, several drugs and substances that target p66^Shc^ or its upstream and downstream molecules have shown beneficial effects against the traditional complications associated with diabetes (as reviewed in [70]). In this review, we focused for the first time on the role of p66^Shc^ in the pathogenesis of the emerging complications of diabetes. In particular, we found that p66^Shc^ may be involved in the pathogenesis of diabetes-related neurodegenerative diseases and cognitive disorders (i.e., AD [118,119,120], PD [121,122], and MS [123,124]), diabetes-caused liver diseases (i.e., NAFLD and NASH [44,84,85,86], and liver fibrosis [87,88,89]), various cancers correlated with diabetes (i.e., breast cancer [101,102,103,104,105,106], colon cancer [107,108], and hepatocellular carcinoma [109,110]), and sleep disorders associated with diabetes (OSA [127]) (Figure 1).

Overall, p66^Shc^ hyperactivation may cover a preponderant role in mediating cellular damage in various tissues/organs, representing a key molecular event in both triggering the onset of diabetes (for instance, when the damage occurs in pancreatic beta-cells) and facilitating the onset of diabetes complications over time. Consequently, p66^Shc^ could represent a promising new therapeutic target for the prevention of the onset and progression of diabetes and its emerging complications, with enormous implications for human health. Future perspectives may include both the identification of new drugs/substances able to interfere with the pathway of p66^Shc^ and the development of RNA-based drugs able to modulate p66^Shc^ expression or activation.

## Data Availability

Not applicable.
